# Editorial for the Special Issue−‘HIV Testing, Prevention, and Care Cascade’

**DOI:** 10.3390/tropicalmed7110387

**Published:** 2022-11-18

**Authors:** Chen Zhang, Yu Liu

**Affiliations:** 1 School of Nursing, University of Rochester, Rochester, NY 14642, USA; 2 School of Dentistry and Medicine, University of Rochester, Rochester, NY 14642, USA

Since the early 1980s, HIV/AIDS has been an ongoing public health concern. By 2021, HIV/AIDS had killed more than 40 million people worldwide, and there were 38 million people live with HIV globally. With tremendous efforts from health professionals, policymakers, and community workers, HIV incidence has continued to fall [1]. Strong evidence indicates that the effective implementation of HIV testing, prevention, and care cascade will help us to end the HIV/AIDS epidemic shortly. Starting with HIV testing, if the individual tests negative, through comprehensive counseling and assessment, they are linked to the prevention cascade (i.e., behavioral and biomedical risk-reduction approaches, continuous monitoring and assessment, and maintaining seronegative status). If a person tests positive, they will be guided into the treatment cascade (i.e., diagnosis, linkage to care, receiving treatment, remaining in care, and receiving viral suppression). Research suggests that the cascade can be enhanced if interventions address supply-side demands (i.e., making prevention or treatment programs available, accessible, and affordable), demand-side demands (i.e., awareness, willingness, and intention to adopt the prevention or treatment approaches), and adherence support (i.e., ongoing engagement with the prevention and treatment programs).

In this current Special Issue, we invited researchers from different regions (e.g., United States, Brazil, Russia, China, and sub-Saharan Africa) working with different key groups (e.g., men who have sex with men (MSM), people living with HIV, pregnant women, and elderly individuals) to contribute their work. In total, twelve studies were published in the current Special Issue, including six original research articles, four secondary data analyses, one review paper, and one modeling study. In this Special Issue, we covered various topics using different study designs (e.g., cross-sectional, qualitative study) and methodologies (e.g., stochastic modeling, machine learning algorithm). Specifically, Zhang et al., Liu et al., and Wang et al. assessed the determinants of HIV testing and its corresponding associations among a group of young MSM [2,3,4]. In addition, using health records, Dzinamarira et al. employed a deep and machine learning algorithm to predict HIV status among 1538 MSM in Zimbabwe [5]. Przybyla et al. and Bleasdale et al. assessed key issues among people living with HIV (PLH) in the United States. They found that single and polysubstance use was associated with lower antiretroviral therapy adherence [6], and the dynamic nature of the ongoing COVID-19 pandemic impacted PLW’s engagement with HIV care [7]. Moreover, several researchers evaluated a few key conditions among PLH in this Special Issue. For instance, Severova et al. assessed the medical records of 2,265 HIV-positive patients co-infected with tuberculosis in Russia, and they found that parenteral transmission (69.4%) remains the primary route of HIV transmission among TB-HIV co-infected patients [8]. Similarly, Shah et al. assessed factors associated with the retention of HIV patients on ART care using 51,296 health records in Congo [9]. Furthermore, Lou et al. employed an innovative strategy by applying Bayesian inference to the gene sequences of two main subtypes of HIV to infer the effective reproduction number to trace the history of HIV transmission among 360 patients who are PLW. Using the outcome from the previous step, they established a model to forecast the spread of HIV medication resistance in the future [10]. Besides MSM and PLW, three other studies assessed the association between HIV testing and fetal ultrasound outcomes among pregnant women in Poland [11]; discovered implementation challenges to the successful roll-out of long-acting cabotegravir in sub-Saharan Africa [12], and evaluated the prevalence and vulnerability factors associated with HIV and syphilis in older people in the Brazilian Amazon [13].

In summary, with efforts from researchers, health professionals, and stakeholders worldwide, we will continue to scale-up science-based approaches among key populations. Using innovative behavioral and biomedical treatment and prevention strategies, we will work together towards the goal of ending the HIV epidemic soon.
